# Metachronous Colorectal Carcinomas and Pancreatic Metastasis in Clinically Suspected Lynch Syndrome: An 18-Year Oncologic Course

**DOI:** 10.7759/cureus.109954

**Published:** 2026-05-31

**Authors:** Manana Jikurashvili, Giorgi Narimanishvili, Ilya Gotsadze, Armaz Mariamidze, David Chechelashvili, Mariam Gendzekhadze, Sopiko Matcharashvili, Giorgi Datunashvili, Salome Tchokhuri

**Affiliations:** 1 Pathology, Tbilisi State Medical University, Tbilisi, GEO; 2 Pathology, Pathology Research Centre, Tbilisi, GEO; 3 Oncology, American Hospital Tbilisi, Tbilisi, GEO; 4 Pathology, Pathology Research Center, Tbilisi, GEO; 5 Pathology, Pathology Laboratory CSD Georgia, Tbilisi, GEO; 6 General Surgery, American Hospital Tbilisi, Tbilisi, GEO

**Keywords:** colorectal cancer (crc), immunohistochemistry, lynch syndrome, metachronous colorectal cancer, multidisciplinary management, pancreatic metastasis

## Abstract

Lynch syndrome (LS) is an autosomal dominant hereditary cancer predisposition syndrome associated with colorectal and extracolonic malignancies. Metachronous colorectal carcinomas are well recognized, whereas pancreatic involvement by metastatic colorectal carcinoma is rare and diagnostically challenging. We report the case of a 58-year-old male patient with a strong family history of colorectal carcinoma and an 18-year history of multiple metachronous colorectal malignancies who developed a pancreatic body-tail mass during oncologic surveillance. Histology showed a high-grade malignant epithelial neoplasm with solid/nested architecture and extensive necrosis. Immunohistochemistry (IHC) demonstrated diffuse SATB2 positivity and loss of MLH1/PMS2 expression, with retained MSH2/MSH6.

The findings favored metastatic colorectal carcinoma involving the pancreas in the setting of clinically suspected Lynch syndrome. This case emphasizes the importance of clinicopathologic correlation, cautious interpretation of MMR deficiency, and long-term multidisciplinary surveillance.

## Introduction

Lynch syndrome (LS), previously termed hereditary non-polyposis colorectal cancer (HNPCC), is the most common hereditary colorectal cancer (CRC) syndrome and accounts for approximately 2%-4% of all CRC cases worldwide [1,2]. It is inherited in an autosomal dominant pattern and results from germline pathogenic variants in DNA mismatch repair (MMR) genes MLH1, MSH2, MSH6, and PMS2 or deletions in EPCAM, which lead to epigenetic silencing of MSH2 [3,4]. Defective MMR function results in microsatellite instability-high (MSI-H) and accumulation of somatic mutations, significantly accelerating the adenoma-to-carcinoma sequence compared with sporadic colorectal carcinogenesis [5]. Specifically, this molecular defect compresses the tumor development timeline from the typical 10-15 years observed in sporadic cases to just one to three years in LS patients, while MMR deficiency overall is identified in approximately 15% of all colorectal malignancies.

The lifetime risk of CRC in LS varies according to the underlying gene mutation but may reach 40%-80% for MLH1 and MSH2 carriers, with somewhat lower penetrance observed in MSH6 and PMS2 mutation carriers [6,7]. LS-associated CRCs characteristically occur at a younger age than sporadic tumors and show a predilection for the proximal (right-sided) colon [6]. A defining clinical feature of LS is the high incidence of metachronous CRCs. Data from the Prospective Lynch Syndrome Database demonstrate a substantial cumulative risk of a second primary CRC after initial resection, particularly in carriers of MLH1 and MSH2 mutations [7]. This elevated risk has important surgical implications, as extended colectomy may be considered in selected patients to reduce future cancer risk [8].

Beyond colorectal malignancies, LS is a multisystem cancer predisposition syndrome. The most common extracolonic malignancy is endometrial cancer, with lifetime risks comparable to or exceeding those of CRC in women [6.9]. Increased risks are also observed for ovarian, gastric, small bowel, urothelial, hepatobiliary, and certain other malignancies [6,9]. Consequently, LS requires lifelong, organ-specific surveillance strategies guided by international recommendations, including those of the National Comprehensive Cancer Network (NCCN) [8].

The diagnosis of LS has evolved significantly over the past decades. Initial identification relied on clinical criteria, including the Amsterdam II criteria and the Revised Bethesda Guidelines [10,11]. However, contemporary practice increasingly supports universal tumor screening for MMR deficiency or MSI in all newly diagnosed CRCs, followed by confirmatory germline genetic testing in appropriate cases [8,12]. This strategy improves detection rates and enables tailored surveillance and management for affected individuals and their families.

From a therapeutic standpoint, the molecular phenotype of LS-associated tumors has major clinical implications. MMR-deficient and MSI-H tumors exhibit high tumor mutational burden and increased neoantigen formation, rendering them particularly responsive to immune checkpoint inhibition [13]. Pembrolizumab and other PD-1 inhibitors have demonstrated significant efficacy in advanced MSI-H/deficient MMR (dMMR) CRC and are now incorporated into standard treatment algorithms, including first-line therapy for metastatic disease [13,14].

The present case illustrates the long-term oncologic course compatible with clinically suspected LS, spanning nearly two decades and involving an initial transverse colon carcinoma, a subsequent metachronous right-sided colorectal malignancy, and later development of a pancreatic lesion in which morphology, SATB2 expression, dMMR status, and clinical history favored metastatic colorectal adenocarcinoma. This complex trajectory underscores the aggressive tumor biology, high metachronous risk, and long-term surveillance challenges inherent to LS.

## Case presentation

A 58-year-old male patient was referred for surgical management of a radiologically detected mass involving the pancreatic body and tail. The patient’s family history was highly significant for hereditary malignancy: his mother was diagnosed with CRC at age 38 (deceased at age 49), and his sister was diagnosed with CRC at age 53 (currently in remission 12 years post-right-sided hemicolectomy).

The patient’s extensive oncologic history spans nearly two decades, reflecting the metachronous nature of clinically suspected LS. In 2008, he was diagnosed with transverse colon cancer and underwent a right-sided hemicolectomy. This was followed by a subsequent resection of the transverse colon in 2021 and the completion of eight cycles of adjuvant chemotherapy (oxaliplatin/capecitabine) in 2022. Most recently, in May 2025, the patient underwent a subsequent surgical resection for a new primary colorectal malignancy. Histopathological evaluation of this specimen revealed synchronous colonic tumors: the first was a high-grade, poorly differentiated adenocarcinoma (Grade 3) with a mucinous carcinoma component spanning approximately 10% of the tumor volume (International Classification of Diseases for Oncology, Third Edition (ICD-O-3) code 8140/3); the second was a moderately differentiated adenocarcinoma (Grade 2, ICD-O code 8480/3) [15]. The maximum tumor diameter was 10.5 cm. Detailed staging confirmed a pT3 N1a Mx status, with one out of 37 isolated lymph nodes positive for metastasis (maximum metastatic diameter 3 mm) without extranodal extension. Lymphovascular invasion (LVI1) and perineural invasion (PNI1) were identified, while surgical resection margins were clear (R0). Tumor budding was classified as intermediate (Bd 2). Immunohistochemistry (IHC) analysis of these 2025 tumor cells demonstrated diffuse positivity for CK20 and CDX-2 and negativity for CK7, CD56, and synaptophysin, establishing a confirmed American Joint Committee on Cancer (AJCC) Stage IIIB (C18) gastrointestinal immunophenotype [16].

Diagnostic evaluation

**Figure 1:**
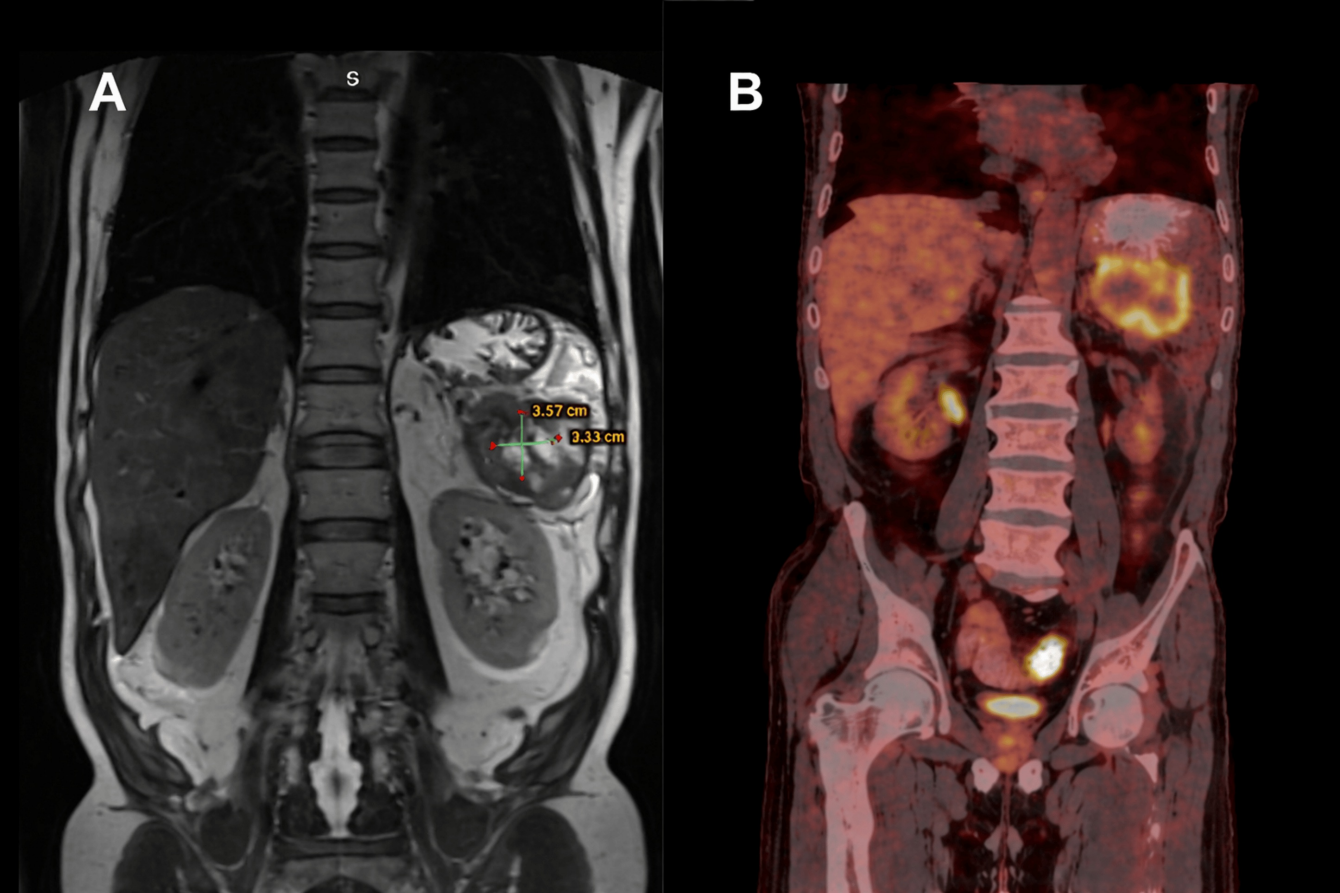
Preoperative radiological imaging A: T2-weighted coronal MRI showing a heterogeneous mass in the projection of the pancreatic tail (measured at 3.57 x 3.33 cm in this plane). B: PET/CT scan demonstrating intense fluorodeoxyglucose (FDG)-avidity (pathological contrast uptake) in the pancreatic tail lesion, consistent with metastatic involvement.

The current pancreatic lesion was discovered incidentally during routine oncologic follow-up. Subsequent MRI identified a mass in the projection of the pancreatic tail measuring 3.57 x 3.33 cm in the coronal plane (Figure 1A), exhibiting heterogeneous diffusion restriction. A PET/CT scan further confirmed pathological fluorodeoxyglucose (FDG) uptake in the same region. To establish the nature of the lesion, an endoscopic ultrasound (EUS)-guided biopsy was performed, which showed metastatic adenocarcinoma, favoring colorectal origin.

Surgical intervention

Radiologically, the mass was noted to involve the pancreatic body and tail, with suspected infiltration into the adjacent gastric wall and the caudal portion of the spleen. The surrounding adipose tissue appeared indurated, accompanied by a small amount of free fluid and radiologic evidence of pathologic lymphadenopathy. Given these findings, the patient underwent a distal pancreatectomy with total splenectomy and regional lymph node dissection. The surgical specimen was then submitted for comprehensive histopathological and immunohistochemical evaluation.

**Figure 2:**
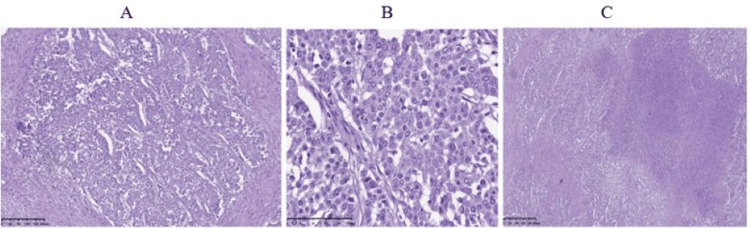
Microscopic examination of the tumor (A) Low-power view demonstrating a predominantly nested growth architecture of the tumor (H&E, ×10). (B) High-power view showing marked nuclear atypia within the solid tumor component (H&E, ×40). (C) Extensive tumor necrosis is present. The combination of solid/nested architecture, significant cytologic atypia, and widespread necrosis supports a high-grade malignant epithelial neoplasm and raises concern for metastatic carcinoma rather than a benign or low-grade pancreatic lesion.

Microscopically, the neoplasm demonstrated predominantly nested and solid architecture, with focal areas showing trabecular and acinar growth patterns (Figure 2A). Tumor cells exhibited round nuclei with granular chromatin and a high nuclear-to-cytoplasmic ratio (Figure 2B). Extensive necrosis was present, with focal comedo-type necrosis (Figure 2C). The mitotic rate was markedly elevated at 36 mitoses per 2 mm². Lymphovascular and perineural invasion were identified. The tumor infiltrated pancreatic parenchyma and splenic tissue, as well as peripancreatic and perisplenic adipose tissue. Although the tumor was closely associated with the gastric wall, and inflammatory granulation tissue was present at the interface, histologic invasion into the gastric wall was not identified.

Ten regional lymph nodes were examined and showed no evidence of metastatic involvement (0/10). All resection margins, including pancreatic, gastric, and circumferential/radial margins, were free of invasive carcinoma, consistent with complete (R0) resection.

Given the high-grade morphology, architectural heterogeneity, and the patient’s prior history of colorectal adenocarcinoma, immunohistochemical studies were performed to further characterize the tumor, determine its origin, and exclude high-grade neuroendocrine carcinoma of the pancreas.

**Figure 3:**
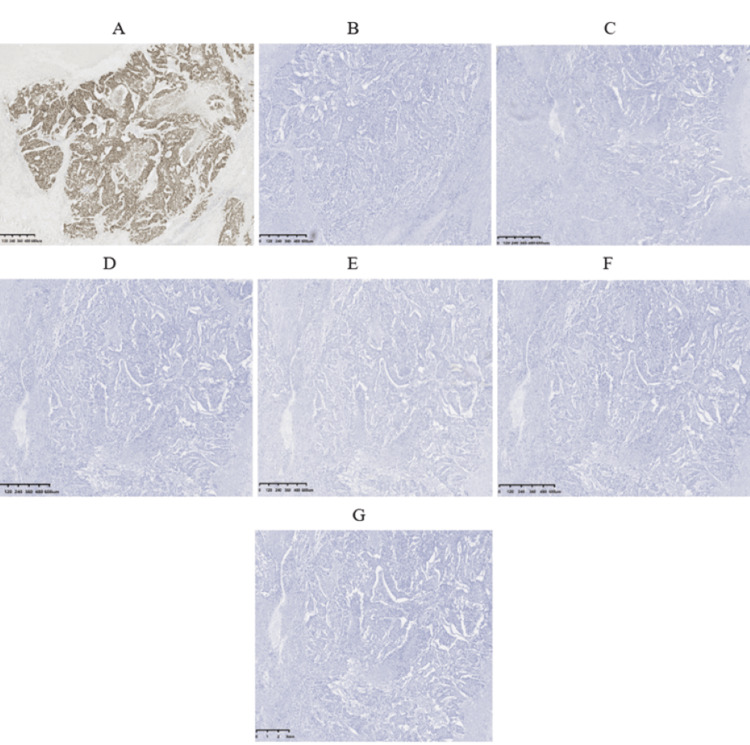
Microscopic examination of the tumor (A) SATB2 immunohistochemical staining demonstrating diffuse nuclear positivity in tumor cells, supporting colorectal origin of the neoplasm (immunohistochemical (IHC)×10). Tumor cells are negative for (B) CD56, (C) CK7, (D) CK20, (E) CDX2, (F) chromogranin, and (G) synaptophysin (IHC ×10). The absence of neuroendocrine markers (CD56, chromogranin, synaptophysin) argues against neuroendocrine differentiation, while the overall immunoprofile, together with SATB2 positivity and clinicopathologic correlation, favored metastatic colorectal adenocarcinoma rather than a primary pancreatic neoplasm.

**Figure 4:**
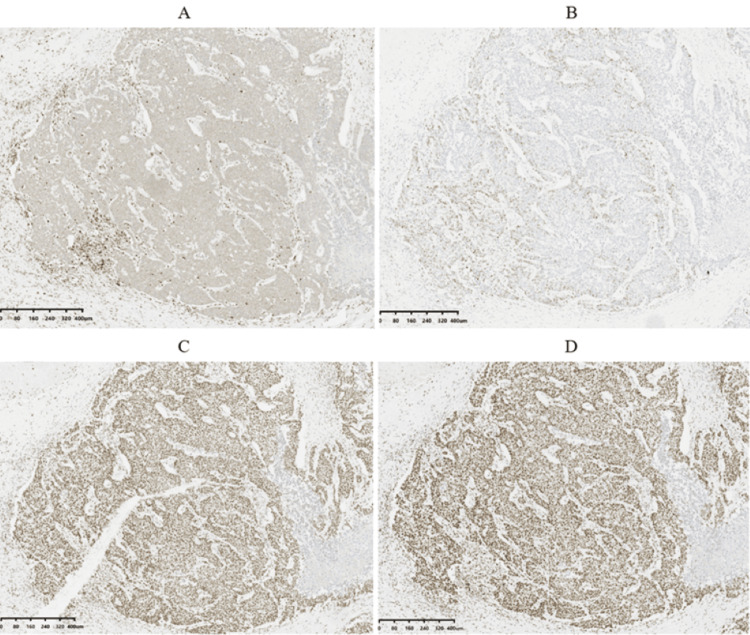
Microscopic examination of the tumor Immunohistochemical staining demonstrates loss of nuclear expression of (A) PMS2 and (B) MLH1 in tumor cells (immunohistochemistry (IHC) ×10), with preserved internal control staining. In contrast, retained nuclear expression of (C) MSH2 and (D) MSH6 is identified (IHC ×10). This mismatch repair-deficient immunophenotype indicates loss of the MLH1/PMS2 protein complex and supports a microsatellite instability-associated carcinoma; however, distinction between sporadic MLH1/PMS2 loss and germline-associated Lynch syndrome requires MLH1 promoter methylation/BRAF testing and/or germline testing.

Tumor cells showed diffuse positivity for AE1/AE3 and SATB2 (Figure 3A) and were negative for CK7, CK20, CDX2, synaptophysin, chromogranin, CD56, and calretinin (Figure 3B-3G). The absence of neuroendocrine marker expression excluded high-grade neuroendocrine carcinoma. MMR protein analysis demonstrated loss of MLH1 and PMS2 (Figures 4A, 4B) expression with retained MSH2 and MSH6 (Figures 4C, 4D), consistent with a dMMR phenotype.

Considering the patient’s history of colorectal adenocarcinoma and the combined morphologic and immunophenotypic findings, the features were most consistent with metastatic colorectal carcinoma involving the pancreas with splenic invasion. Molecular confirmation of MSI was recommended.

Chronological summary of the clinical course

To provide a clear overview of the patient’s complex 18-year oncologic history, the key events are summarized chronologically as follows: 2008: Diagnosis of transverse colon adenocarcinoma treated with right-sided hemicolectomy; 2021: Metachronous recurrence in the transverse colon; treated with subsequent resection and eight cycles of adjuvant oxaliplatin/capecitabine (completed in 2022); 2025 (May): Synchronous primary colorectal adenocarcinomas (Grade 3 poorly differentiated mucinous and Grade 2 moderately differentiated, Stage IIIB, CK20+/CDX-2+/CK7-); treated with left-sided hemicolectomy; 2026 (current): Metastatic colorectal adenocarcinoma involving the pancreatic body and tail with splenic invasion; confirmed by SATB2(+) and dMMR profile (loss of MLH1/PMS2). Treated with R0 distal pancreatectomy and splenectomy.

## Discussion

LS is an autosomal dominant hereditary cancer predisposition syndrome caused by constitutional pathogenic variants affecting the DNA MMR genes MLH1, MSH2, MSH6, and PMS2 or, less commonly, EPCAM alterations that lead to epigenetic silencing of MSH2 [3,8]. Defective MMR function results in MSI and accumulation of somatic mutations, which accelerate tumorigenesis and predispose affected individuals to a wide spectrum of malignancies [4,12]. Although historically referred to as hereditary non-polyposis CRC, the syndrome is now recognized as a multisystem cancer predisposition disorder involving not only the colorectum but also the endometrium, ovary, stomach, small bowel, hepatobiliary tract, urinary tract, pancreas, brain, and other organs [6,9].

The present case illustrates one of the most characteristic clinical features of LS: the development of metachronous colorectal carcinomas. Patients with LS remain at substantial risk for additional primary CRCs even many years after initial tumor resection. Data from the Prospective Lynch Syndrome Database demonstrate a significant cumulative risk of second colorectal malignancies over time, particularly in carriers of MLH1 and MSH2 pathogenic variants [7]. The prolonged 18-year oncologic course observed in the present patient is therefore consistent with the well-documented natural history of LS.

Another notable aspect of this case is the occurrence of a pancreatic mass representing metastatic colorectal adenocarcinoma. Pancreatic involvement in LS presents a complex diagnostic scenario because pancreatic tumors may arise as primary LS-associated malignancies or represent metastatic disease from another LS-related cancer. Metastases to the pancreas are uncommon overall and account for approximately 2% of pancreatic malignancies, with the most frequent primary sites being renal cell carcinoma, lung carcinoma, melanoma, and breast carcinoma [17]. Metastatic spread from colorectal carcinoma to the pancreas is particularly rare and is reported only sporadically in the literature [18,19]. Therefore, accurate identification of tumor origin requires careful integration of morphologic, immunohistochemical, and clinical findings.

Histologically, colorectal carcinomas associated with MSI frequently demonstrate distinctive features, including tumor-infiltrating lymphocytes, a Crohn-like peritumoral lymphoid reaction, poor differentiation, mucinous or signet-ring cell morphology, and a medullary growth pattern [20-24]. However, these findings are observed in both sporadic MSI-high tumors and LS-associated carcinomas and are not sufficiently specific to reliably distinguish microsatellite-stable from microsatellite-unstable tumors on morphologic grounds alone. This limitation is particularly relevant in metastatic lesions or tumors arising at unusual anatomic sites.

In the present case, the pancreatic lesion demonstrated predominantly solid and nested architecture with focal trabecular and acinar-like growth, extensive necrosis, and a markedly elevated mitotic rate. Such morphologic features may overlap with several primary pancreatic neoplasms, including high-grade neuroendocrine carcinoma, poorly differentiated ductal adenocarcinoma, or acinar cell carcinoma. Interestingly, certain pancreatic tumors reported in association with LS include medullary carcinoma and acinar cell carcinoma, which may show MSI and MMR deficiency [25,26]. Consequently, histomorphology alone was insufficient to establish tumor origin.

In this setting, IHC played a crucial diagnostic role. Diffuse expression of SATB2, a transcription factor strongly associated with colorectal epithelial differentiation, provided important supportive evidence for colorectal origin [20]. While the immunohistochemical profile of typical colorectal adenocarcinoma usually follows a CK7-/CK20+/CDX2+ pattern, it is important to recognize that MSI-H/dMMR tumors, particularly those with medullary or highly undifferentiated features, frequently deviate from this classic immunophenotype. The loss of traditional markers like CK20 and CDX2 is a recognized phenomenon in LS-associated cancers as they become more undifferentiated. In this case, the CK7-/CK20- profile, while unusual for a typical CRC, is compatible with the aggressive, poorly differentiated morphology of a long-standing dMMR clone. This diagnostic 'decoy' further underscores the necessity of using broad-spectrum markers like SATB2 to support intestinal origin when standard markers fail.

MMR IHC further supported the diagnosis. The tumor demonstrated loss of MLH1 and PMS2 expression with retained MSH2 and MSH6, indicating defective MLH1 function. This pattern reflects the heterodimeric structure of the MMR protein complexes, in which MLH1 forms a functional pair with PMS2; loss of MLH1 therefore leads to secondary loss of PMS2 expression [12]. Immunohistochemical screening for MLH1, PMS2, MSH2, and MSH6 has become a widely accepted first step for detecting MMR deficiency in colorectal carcinomas. The presence of all four proteins suggests MIS, whereas the loss of nuclear staining for any protein indicates MMR deficiency and guides further genetic evaluation.

Nevertheless, interpretation of MMR IHC requires awareness of potential pitfalls. Technical factors such as uneven fixation or antibody penetration may produce patchy staining, while cytoplasmic staining without nuclear expression should be interpreted as abnormal [27,28]. In addition, approximately 3%-10% of LS-associated tumors with MSI may show apparently intact immunohistochemical expression, likely due to mutations that impair protein function without affecting antigen detectability [29]. Therefore, IHC and MSI testing should be interpreted in conjunction with clinical and molecular findings.

Loss of MLH1/PMS2 expression may occur either in the context of LS or in sporadic tumors due to MLH1 promoter hypermethylation, which is often accompanied by the BRAF p.V600E mutation [12]. Accordingly, current diagnostic algorithms recommend additional molecular testing, including BRAF mutation analysis or MLH1 promoter methylation studies, to distinguish sporadic MSI-high tumors from LS-associated cancers [8]. In the present case, the strong family history and the occurrence of metachronous colorectal carcinomas strongly suggest an inherited MMR deficiency, although definitive confirmation requires germline genetic testing.

Beyond its diagnostic value, mismatch repair deficiency also has important therapeutic implications. MSI-H tumors exhibit a high tumor mutational burden and increased neoantigen formation, making them particularly susceptible to immune checkpoint blockade [4,13]. Clinical trials have demonstrated the substantial efficacy of PD-1 inhibitors, including pembrolizumab, in patients with MSI-high metastatic CRC, and these agents are now incorporated into standard treatment algorithms [13,14].

Finally, this case highlights the importance of lifelong surveillance in individuals with LS. Because LS confers a persistent risk of both colorectal and extracolonic malignancies, current clinical guidelines recommend regular colonoscopic monitoring and risk-adapted surveillance for other associated cancers [6,8]. The incidental detection of the pancreatic lesion during routine follow-up in the present patient underscores the value of long-term vigilance and multidisciplinary management in hereditary cancer syndromes.

In summary, this case illustrates the complex natural history of LS, characterized by metachronous colorectal carcinomas and rare metastatic spread to the pancreas. It also emphasizes the importance of integrating morphology, IHC, molecular diagnostics, and clinical context to establish tumor origin and guide patient management.

This case report has several limitations. Molecular testing was limited, including in the previously resected colorectal carcinoma specimens and other prior operative materials. PCR-based confirmation of microsatellite instability was not performed. In addition, MLH1 promoter methylation analysis and BRAF mutation testing were unavailable. These ancillary molecular studies would be important to further distinguish sporadic MMR deficiency from a germline-associated mismatch repair deficiency consistent with Lynch syndrome. Therefore, interpretation of the MMR-deficient phenotype in this case relies predominantly on the clinical history, histopathologic findings, and immunohistochemical profile.

## Conclusions

This case highlights the complex, long-term oncologic course of clinically suspected LS, characterized by metachronous malignancies and rare metastatic events. The diagnostic challenge posed by the pancreatic lesion underscores the importance of recognizing that dMMR/MSI-H tumors often lose classic immunohistochemical markers (CK20/CDX2) as they undergo dedifferentiation. The use of SATB2 proved pivotal in supporting the colorectal origin of the metastasis and favored a metastatic colorectal origin in the appropriate clinical context. Ultimately, this 18-year history emphasizes that for patients with clinically suspected LS, vigilant long-term surveillance and a multidisciplinary approach are essential to managing the persistent risk of both local recurrence and atypical systemic spread.
